# Case Reports in Oncological Medicine Myoepithelioma: A New Rearrangement Involving the *LPP* Locus in a Case of Multiple Bone and Soft Tissue Lesions

**DOI:** 10.1155/2018/3512847

**Published:** 2018-02-27

**Authors:** Géraldine Pairet, Gaëlle Tilman, Rafaël Sciot, Thomas Schubert, Vasiliki Perlepe, Hélène A. Poirel, Christine Galant

**Affiliations:** ^1^Department of Pathology, Cliniques Universitaires Saint-Luc, Brussels, Belgium; ^2^Center for Human Genetics, Cliniques Universitaires Saint-Luc–Université catholique de Louvain, Brussels, Belgium; ^3^Department of Pathology, Katholieke Universiteit Leuven, Leuven, Belgium; ^4^Department of Orthopaedic Surgery, Cliniques Universitaires Saint-Luc, Brussels, Belgium; ^5^Department of Medical Imaging, Cliniques Universitaires St-Luc, Brussels, Belgium; ^6^Center for Human Genetics, Cliniques Universitaires Saint-Luc and Human Molecular Genetics, de Duve Institute, Université catholique de Louvain, Brussels, Belgium; ^7^Department of Pathology, Cliniques Universitaires St-Luc and IREC, Pôle de morphologie MORF, Université Catholique de Louvain, Brussels, Belgium

## Abstract

We report a case of multiple myoepithelioma with synchronous bone and soft tissue tumors, associated with a new genomic alteration of the *LPP* locus. The lesions occurred in the foot by presenting one lump in the plantar soft tissue, and three lesions were detected in the calcaneus and in the navicular bone. All tumors showed the double immunophenotype of epithelial markers and S100 protein expression. No rearrangement of the *EWSR1* and *FUS* loci was detected as reported in myoepitheliomas. However, molecular karyotyping detected an unbalanced rearrangement of the *LPP* locus, not involving the *HMGA2* locus, which is the most frequent translocation partner observed in benign mesenchymal tumors such as lipomas (of soft tissue as well as parosteal) and pulmonary chondroid hamartoma.

## 1. Introduction

Myoepithelial tumors (METs) of soft tissue and bone are rare tumors of uncertain histogenesis. The first deep tumor was described in the retroperitoneum [1], followed by a large series in 1997 including cases in soft tissue [2]. Rare cases in bone are more recently reported, and these lesions tend to occur in the acral region of the lower limbs usually in middle age male patients [3–7]. Axial localization of METs has to be distinguished from chordoma.

Histologically, METs are made of a homogeneous population of myoepithelial cells. They could be considered as a part of a continuum with mixed tumors when ductal differentiation is present. They may harbor chondroid and bone differentiation as observed in classical mixed tumors. The diagnosis of MET requires the coexpression of both epithelial markers and S100 protein [8, 9]. They share morphological and immunohistochemical features with their counterparts described in skin and salivary gland.

A different genetic pattern distinguishes METs arising in the skin from those in deep soft tissue and bone. *EWSR1*(22q12) gene fusions have been detected in half of METs arising in deep soft tissues and in up to 70% cases of intraosseous MET [7, 10]. Several partners of *EWSR1* are described: *POU5F1*(6p21.33) (16%), *PBX1* (16%), *PBX3*, *ZNF444*, *ATF1*, *KLF17*, and *NFATC2* [10–16]. The *EWSR1-POU5F1* more often occurs in children and young adults while the *EWSR1-PBX1* occurs in middle-aged adult patients [11, 14]. The tumors of the first subgroup show a solid or nested growth arrangement of tumor cells showing at least partially a clear appearance of the cytoplasm. The subgroup with *EWSR1-PBX1* rearrangement presents a bland sclerotic appearance or clear cell morphology with a diffuse EMA staining. However, none of the *EWSR1*-rearranged tumors show the presence of ductal or glandular differentiation or cartilage/bone matrix formation [10]. Rearrangement of the *FUS*(16p11) gene has also been reported in rare cases of MET arising in deep soft tissue as well as in bone [15, 17]. Two gene partners have been characterized, *KLF17* and *POU5F1*. These *FUS*-rearranged tumors also lack ductal differentiation [15].

*PLAG1* (8q12.1) and *HMGA2* (12q14.3) rearrangements are the most common genetic events in pleomorphic adenomas [18]. *PLAG1* is also found in MET [19, 20]. In the study of Anthonescu et al., 3 cutaneous and 10 soft tissue METs (out of a total of 35 tumors) showed the presence of *PLAG1* gene rearrangement [19]. All tumors except one showed tubular differentiation, suggesting that MET with tubuloglandular differentiation, called mixed tumors of skin or of soft tissue, are genetically linked to their salivary gland counterpart.

We report an unusual observation in a 52-year-old man of a multifocal MET without obvious ductular differentiation and harboring a new *LPP* unbalanced rearrangement without *EWSR1* and *FUS* alterations in both soft tissue and bone lesions.

## 2. Case Presentation

**Figure 1:**
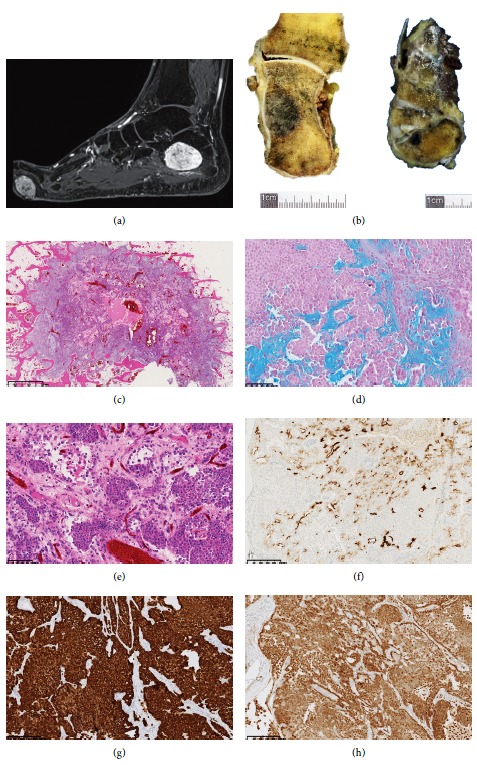
Characterization of the lesions on MRI, macroscopic, microscopic, and immunohistochemical level. (a) MRI gadolinium-enhanced fat-saturated T1-weighted image in the sagittal plane showing an intraosseous lesion of the calcaneus and a plantar soft tissue lesion of the forefoot (arrows). (b) Macroscopic appearance of lesions in the calcaneus and in the cuneiform bone (arrows). (c–e) Lobulated infiltrative pattern made of plasmacytoid cells (H and E). (d) Staining of the chondromyxoid stroma by alcian blue. (f–h) Focal expression of the tumor cells for EMA (f), diffuse expression for cytokeratin AE1/AE3 (g), and diffuse expression for S100 protein (h). Sections of bone were performed using the diamond band saw (EXAKT312, Germany) and decalcified with a formamid solution (DC1, V.W.R.) after formalin fixation. 5 µm thick sections of the paraffin-embedded material were stained with H and E (Symphony 5-Plus, Roche). Immunohistochemistry experiments were performed according to standard procedures. Primary antibodies used on XT benchmark platform (Ventana) were CKAE1/AE3 (cloneAE1/AE3; 1.8 mg/L), EMA (clone E29; 2.4 mg/L), protein S100 (rabbit polyclonal; 1/100), GFAP (rabbit polyclonal; 1/500), alpha-smooth muscle actin (clone 1A4; 0.4 mg/L), desmin (clone D33; 2.05 mg/L), INI1/BAF47 (clone 25/BAF47; 2.5 mg/L), and vimentin (clone V9; 0.5 mg/L).

A 52-year-old patient complained of pain and swelling of the foot. MRI and plain radiography demonstrated a main lesion in the calcaneus, two others in the navicular bone, and a last one in the plantar soft tissue (Figure 1). The main bone lesion was first investigated by fine needle aspiration. The soft tissue lesion was then resected.

### 2.1. Pathology

The tumor of the calcaneus measured 4.4 × 3.4 cm (Figure 1), and the other lesions in the medial cuneiform measured 2.2 × 0.9 cm (Figure 1) and 0.5 mm. The one in the soft tissue, resected independently, measured 3.5 × 2.7 cm. On histology, the different tumors appeared lobulated and contained plasmacytoid cells arranged in lobules and in large cellular sheets, intermixed with areas of fibrous and chondromyxoid stroma positive for alcian blue (Figure 1). These cells showed mild nuclear pleomorphism and some cysts were observed, but no convincing ductular differentiation (Figure 1). Some groups of tumor cells were detected in peripheral vascular spaces, without obvious fibrin but suspicious for vascular emboli (not shown).

Immunohistochemistry demonstrated an intense and diffuse staining of plasmacytoid cells for the broad-spectrum cytokeratin, S100 protein (Figures 1 and 1), and vimentin. A focal staining for GFAP was noticed. EMA was focally positive in the cytoplasm, and a very focal membranous apical staining was also present (Figure 1). There was a diffuse nuclear staining for INI1. Alpha-smooth muscle actin and desmin immunostainings were negative.

These results were in favor the diagnosis of a MET. Based on the multiplicity of localizations, a transtibial amputation was decided by the local multidisciplinary committee. Although lesions were multiple and some pictures were suspicious for vascular emboli, the patient had no recurrence or distant metastasis 2 years later. The last follow-up detected small lung lesions, which remained stable and were considered as aspecific.

### 2.2. Cytogenetics

**Figure 2:**
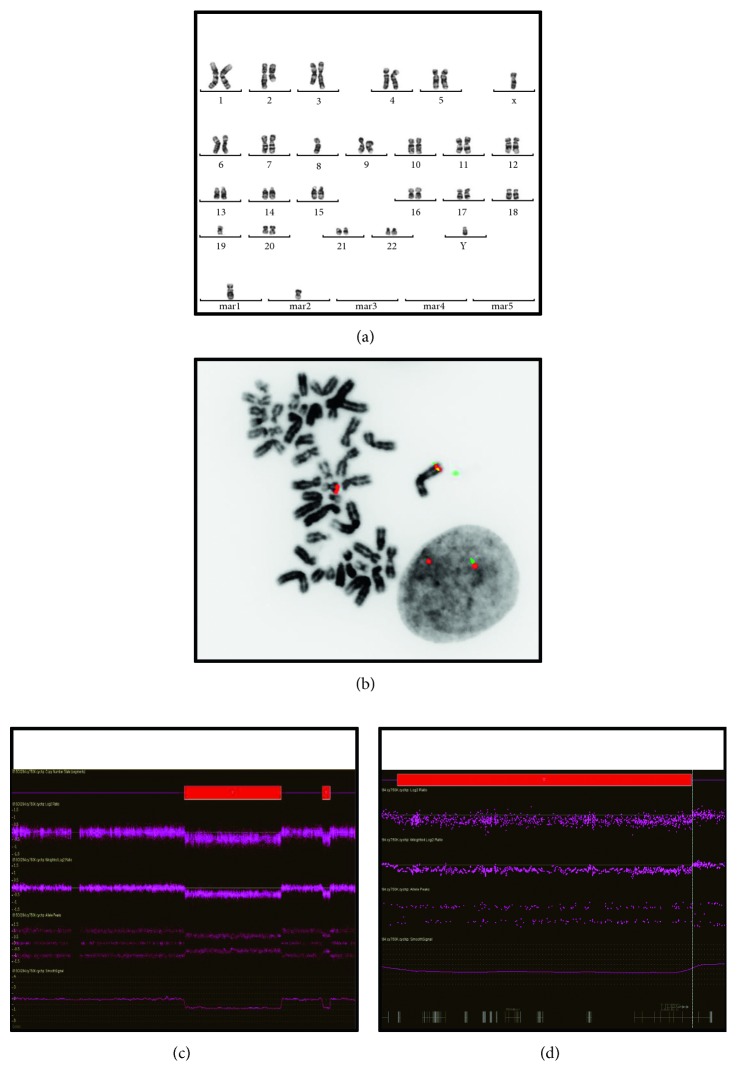
Cytogenetic characterization. (a) Conventional karyotype on the bone tumor (calcaneum): 46,XY,add(2)(q?21),der(3)t(2;3)(q?21;q?21),-8,-19,+mar1,+mar2[8]. The soft tissue tumor harbor the same chromosomal aberrations: 46,XY, add(2)(q?21),der(3)t(2;3)(q?21;q?21),-8,-19,+mar1,+mar2[2]. (b) FISH experiments with break-apart probes on fixed cells: deletion of the BAC probe located 5'/centromeric (RP11-1144D2 labelled in green) to the *LPP* (lipoma preferred partner or LIM Domain Containing Preferred Translocation Partner In Lipoma) locus in a metaphasic cell and in a nucleus (soft tissue tumor): ish der(?)t(3,?)(RP11-1144D2-,RP11-67E18-;RP11-67E18+[5].nuc ish(RP11-1144D2x1,RP11-67E18x2)(RP11-1144D2 con RP11-67E18x1)[334/400]. (c, d) Molecular karyotyping (soft tissue tumor): 2 interstitial deletions within the long arm of chromosome 3 (c) and the telomeric one delete the 5' part of the *LPP* locus (d) arr[hg19] 3q22.1q26.2(133,374,187–169,925,119),3q27.2q28(185,628,780-188,411,171)x1. Culturing, harvesting, and G-banding of the tumor samples for karyotyping were performed according to standard procedures [21]. Culturing, harvesting, and G-banding of the tumor samples for karyotyping were performed according to standard procedures [21]. Dual-color FISH experiments were performed on fixed nuclei and on formalin-fixed paraffin-embedded tissue sections (4 *μ*m-thick), using commercial probes (LSI-*EWSR1*, LSI-*FUS*, LSI-TP53/CEP17, LSI-9p21/CEP9, LSI-TP53/CEP17 from Abbott Molecular/Vysis; ON-TFE3 from Kreatech) and bacterial artificial chromosome (BACs) probes. The BAC clones were purchased from the Chori BACPAC Resources Center (Oakland, USA) to study the following loci: NR4A3/9q31.1 (RP11-412F16, RP11-47M15, RP11-266D8, and RP11-282C24); *HMGA2*/12q14.3 (RP11-317J13, RP11-412I20, RP11-945G8, and RP-347J7); and *LPP*/3q27.3-q28 (RP11-1144D2 and RP11-67E18). Extraction, labeling, and hybridization were performed, as previously reported [22]. Two hundred interphasic cells and all hybridized metaphases were analysed. Molecular karyotyping was performed with Cytoscan 750K SNP-arrays according to the manufacturer's instructions (Affymetrix). Results were analysed as previously reported [23]. Aberrations greater than 100 kb involving at least 20 consecutive SNPs were considered for copy number variant (CNV) analysis. Constitutional CNV polymorphisms were excluded based on comparisons with the Database of Genome Variants (hg19). The quality control metrics were within the normal range (SnpQC = 17.656, Mapd = 0.195, and Waviness = 0.088).

Conventional karyotyping detected the same abnormal complex pseudodiploid clone in the soft tissue tumor as in the bone tumor (Figure 2).

FISH experiments were performed to look for rearrangement of genes known to be altered in MET arising in deep soft and bone tissues, *EWSR1* and *FUS*. FISH detected no rearrangement of those loci. Other differential diagnoses were excluded by FISH: extraskeletal myxoid chondrosarcoma (*NR4A3*) and alveolar soft part sarcoma (*TFE3*).

Molecular karyotyping detected 2 main deletions on the long arm of chromosome 3 located at 3q22.1-3q26.2 (37Mb) (Figure 2) and 3q27.2-3q28 (3Mb). The latter includes the 5' part of the *LPP* gene, probably the 8 first exons (Figure 2). Three other interstitial deletions were identified at 8p22 (3Mb), 8q23.3 (0.3Mb), and 13q14.3 (1.4Mb).

FISH experiments with BAC probes confirmed the 3q27.3-q28 unbalanced rearrangement with the loss of the probe located 5'/centromeric to the *LPP* locus (Figure 2) but did not detect any rearrangement of the *HMGA2* locus which is the most frequently reported partner gene of *LPP* in different benign mesenchymal tumors.

## 3. Discussion

MET can occur in various sites, but only 9% of MET occurs in bone. Most of the deep-seated lesions in bone are incidental discoveries. Tumor sites are tibia, ilium, vertebra, maxilla, and sacrum [24–26]. As far as we know, only a cutaneous and subcutaneous MET has been reported in the foot [27]. We described here synchronous tumors in two different bones of the foot and in the soft tissue of a 52-year-old man with no further aggressive evolution.

This observation is also unusual at the genetic level. We did not detect the rearrangement of the *EWSR1* and *FUS* genes classically involved in MET arising in deep soft and bone tissue without ductal differentiation. Instead, a hitherto unreported rearrangement of the *LPP* locus was found. The *LPP* gene is known to be rearranged through chromosomal translocations [28]. The most frequent one is the t(3;12)(q27-q28;q14-q15), which is recurrent in lipomas (of soft as well as parosteal tumor) and fuses *LPP* with *HMGA2* [29]. This translocation is also described in pulmonary chondroid hamartomas [30] and in one case of soft tissue chondroma [31].

Three other *LPP*-partner genes have been reported in one case of lipoma, *HMGA1*(6p21) [32], and in 2 different hematological malignancies: *KMT2A*(11q23) in a secondary acute leukemia [28] and *BCL6*(3q27) through a 3q27 interstitial deletion in a primary central nervous system lymphomas [33].

The oncogenic role of *LPP* remains unclear, while the role of the partner gene seems to be crucial. Several lines of evidence suggest that *HMGA2* truncation occurring in the most common t(3;12) fusion gene is implicated in lipomagenesis. However, the *HMGA2-LPP* fusion protein retains the transactivation functions of two *LPP* LIM domains which might contribute to the mesenchymal tumorigenesis by directly affecting transcriptional regulation processes [34].

In the present case, the suspected breakpoint within the *LPP* locus suggests that carboxy-terminal LIM transactivating domains may contribute to the chimeric protein with the aminoterminal part of an unidentified gene. Although not rearranged by FISH, we cannot exclude a cryptic insertion of the 5' part of *HMGA2* within the *LPP* locus. The only candidate locus for a gene fusion with *LPP* may be *TRPS1* (8q23.3) whose 3' part is deleted. TRPS1 is known to be associated with tumorigenesis, metastasis, and angiogenesis in several tumors, including osteosarcoma [35]. Unfortunately, it was not possible to further characterize the *LPP*-partner gene by 3' RACE-PCR or RNASeq.

No ductal differentiation was present although membranous EMA staining was focally detected. The stroma was focally myxoid but without a mesenchymal cell population as observed in mixed tumors. Recurrent genetic abnormalities involving *PLAG1* and *HMGA2* have been described in pleomorphic adenomas (mixed tumors) of salivary gland [36]. *PLAG1* rearrangements were mainly identified in a subset of cutaneous and superficial soft tissue MET tumors, often displaying ductal structures and considered as mixed tumors [10]. *PLAG1* has not been tested because of the absence of ductular differentiation in the tumor.

We describe here a new rearrangement of the *LPP* (3q27-3q28) locus in synchronous tumors presenting epithelioid features. The partner gene remains to be characterized. Analysis of the *LPP* locus should be performed by FISH on MET without *EWSR1* or *FUS* rearrangements and pleomorphic adenomas of salivary gland without *PLAG1* and *HMGA2* aberrations to define the recurrence and the tumor characteristics associated with this new alteration.
